# Attitudes among parents of persons with autism spectrum disorder towards information about genetic risk and future health

**DOI:** 10.1038/s41431-021-00966-y

**Published:** 2021-11-15

**Authors:** Jarle Johannessen, Terje Nærland, Sigrun Hope, Tonje Torske, Anett Kaale, Katrine V. Wirgenes, Eva Malt, Srdjan Djurovic, Marcella Rietschel, Ole A. Andreassen

**Affiliations:** 1 K. G. Jebsen Centre for Neurodevelopmental Disorders, Oslo, Norway; 2 NevSom, Department of Rare Disorders, Oslo University Hospital, Oslo, Norway; 3 Division of Mental Health and Addiction, Vestre Viken Hospital Trust, Drammen, Norway; 4 Department of Special Needs Education, University of Oslo, Oslo, Norway; 5 Department of Medical Genetics, Oslo University Hospital, Oslo, Norway; 6 Institute of Clinical Medicine, Campus Ahus, University of Oslo, Oslo, Norway; 7 Department of Adult Habilitation, Akershus University Hospital, Nordbyhagen, Norway; 8 NORMENT, Department of Clinical Science, University of Bergen, Bergen, Norway; 9 Department of Genetic Epidemiology in Psychiatry, Central Institute of Mental Health, Faculty of Medicine Mannheim, University of Heidelberg, Heidelberg, Germany; 10 NORMENT, Institute of Clinical Medicine, University of Oslo, Oslo, Norway; 11 Division of Mental Health and Addiction, Oslo University Hospital, Oslo, Norway

**Keywords:** Genetic counselling, Ethics, Medical ethics

## Abstract

Clinical relevance of genetic testing is increasing in autism spectrum disorder (ASD). Information about genetic risk may contribute to improved diagnostics, treatment and family planning, but may also be perceived as a burden. Knowledge about the families’ preferences with regard to genetic risk information is important for both health care professionals and policy makers. We investigated attitudes towards sharing information about genetic risk of ASD and knowledge about future health among parent members of the Norwegian Autism Association (*N* = 1455) using a questionnaire, and the relationships with parent and child characteristics, such as age, gender and ASD severity. Most preferred autonomy in deciding whom to inform about genetic risk of ASD (74.4%) and a minority supported extensive intra-familial disclosure of the genetic risk (41.1%). The majority agreed that it is an obligation to know as much as possible relevant for future health (58.0%) and only 51.7% agreed to a principle of a ‘right not to know’. In regression models, the attitudes were associated with opinions about benefits and harms of genetic testing (e.g., treatment, family planning, understanding of ASD pathology, insurance discrimination and family conflict). In sum, the findings show that most parents want to know as much as possible relevant for their children’s future health and keep their autonomy and intra-familial confidentiality about genetic risk information. Nearly half of the parents were not concerned with a “right not to know”. These attitudes can inform development of guidelines and bioethics in the age of genomic precision medicine.

## Introduction

Autism spectrum disorder (ASD) is defined by social communication and interaction deficits and repetitive behaviours. The global prevalence is about 1% and disease burden is high [1]. Heritability is estimated up to 90% [2]. Consistent with complex inheritance, both environmental risk factors and common gene variants with individually small effect size have been implicated in disease etiology [3, 4]. Rare variants or copy number variants, especially de novo variants, associated with a large risk of ASD are found in a minority of persons [5]. The relative contribution of rare and common variants has been estimated to 2.5–15% and 12–52%, respectively [6].

Genetic tests have shown little practical benefit for most people with ASD [5]. An ASD diagnosis is still based on descriptive and behavioural criteria [7]. The proportion of cases in which the etiology can be determined with genetic testing is expected to increase to 30–40%, and clinical use is expanding [8]. Clinical genetic testing may have potential to aid early identification, predict prognosis and guide interventions [9]. It may also trigger screening for medical problems, provide a medical explanation and help find specific support groups and assist family planning [5, 10].

Genetic testing typically includes extensive counseling and emotional support. Still, the process of absorbing genetic risk information may be difficult for parents. Identity issues may arise, [11] genetic contributions may be overestimated and consequently the value of behavioural interventions could be downplayed [12]. Family planning may be influenced in form of discontinued reproduction [13] and issues of blame and guilt [14] may affect family relations [15]. Stigmatization [16], discrimination in employment and insurance [17] and negative general attitudes in society at large may also occur [18, 19]. For non-psychiatric disorders, no large negative, ethical, legal and social impacts (ELSI) of clinical genetic testing have been identified in the general population [20]. However, impacts may vary across traits investigated and of the test results [20, 21].

To reduce negative impact of genetic risk information a “*right not to know*” may be claimed. This right has support in various ethical and legal instruments, e.g., the European *Convention on Human Rights and Biomedicine*, UNESCO’s *Universal Declaration on the Human Genome and Human Rights* [22] and the World Health Organization’s *Review of Ethical Issues in Medical Genetics* from 2003. A “*right not to know”* ‘may be in opposition to e.g., the ‘*right to know’* and health care professionals’ obligation to inform, and these principles [23] are usually balanced on a case to case basis. Little is known about attitudes towards these principles in parents of children with ASD, making it difficult to develop empirically informed ethical guidelines on genetic testing and the return of genetic results in clinical practice [23].

In general psychiatry the majority of patients and parents have claimed full autonomy in receiving and sharing genetic risk information and full support of professional-patient confidentiality [24, 25]. In the present study we focus on genetic testing in ASD. Our aim was to explore parents’ attitudes towards four aspects: (i) Should parents decide whom to inform about genetic risk information? (ii) Should genetic risk information be shared extensively in the family? (iii) Do we have an obligation to obtain information about future health? and (iv) How important is a ‘*right not to know’*? We also investigated how the responses to these attitude questions were related to characteristics of parent (i.e., age and gender) and child (i.e., ASD severity and gender) relevant for clinical genetic testing and counseling. To make the findings relevant for practical bioethical guidelines, parents in favour and parents against clinical genetic testing for ASD were separated in the analyses. Finally, we explored the relation between parents’ attitudes and previously reported data on perceptions of benefits and harms of clinical genetic testing in regression models [26]. Based on previous findings in general psychiatry [24, 25, 27] we have four hypotheses: (#1) The majority of parents want to decide themselves whom to inform about genetic risk, independent of their child’s disease severity (Asperger syndrome vs. infantile autism); (#2) The majority of parents prefers no intra-familial disclosure of genetic risk; (#3) The majority of parents agree that everyone has an obligation to know as much as possible relevant information for future health of themselves and their children and that this opinion will be most wide-spread in parents of children with severe ASD; (#4) The majority of parents find a ‘*right not to know*’ about health information important, and this attitude is particularly prevalent among parents with children with milder ASD.

## Materials and methods

### Participants

In collaboration with the Norwegian Autism Association, all parent members were invited to participate. Those registered with email addresses were contacted by email, which included a link to a web-based questionnaire. Parents without a registered email address received the questionnaire by paper mail, which included a prepaid return envelope. Email but not paper mail reminders were sent to those not responding. Answers were anonymous. In total, 3539 invitations were sent (1990 by email and 1549 by paper mail) and 1455 parents responded (41%). Of those, 917 (63%) responded through the web and 538 (37%) by posting the questionnaire. Eleven responses were missing parents’ age, 7 were missing parents’ gender, 23 were missing children’s age, 29 were missing children’s gender and 39 were missing specific ASD diagnosis. No financial incentive or other rewards were provided to the respondents. The survey was open to responders from first half of 2014 and closed at the end of 2014 after two reminders.

### Norwegian setting

This study was conducted in Norway where WHO’s International Classification of Diseases 10th revision (ICD-10) has been used since 1997. Norway has a public health and school system. Those with special needs have the right to individually tailored special education services. The most common intervention programs for individuals with ASD are applied behaviour analysis (ABA) or ABA derivatives, various forms of augmentative and alternative communication and the TEACCH method [28]. The public health care system provides genetic testing, centralized to medical genetics departments at the university hospitals. If genetic test is indicated, the first-tier diagnostic genetic test is array CGH (CNV screening) or specific mutation screening [3]. If the condition is complicated by intellectual disability or congenital malformations, exome sequencing with parental samples might be offered. When a CNV or rare variant is identified in a child with ASD, the family is offered genetic counseling and offered predictive testing/carrier testing of the parents.

### Questionnaire

The questions concerning attitudes to genetic testing were part of a questionnaire with 45 items divided into four sections. The first section asked about age and gender of the parents. The second section asked about the parent’s firstborn child with an ASD diagnosis, the child’s specific ASD diagnosis (infantile autism or Asperger syndrome), age at diagnosis, comorbid somatic and psychiatric diagnoses and services offered and received from the specialist health care system. Parents were not asked how many children they have with ASD. The fourth section asked 23 questions about attitudes towards (i) genetic research, (ii) clinical genetic testing and (iii) receiving information on general health related information and ASD-specific genetic risk. The questionnaire emphasized that a predictive genetic test for ASD is currently not available, but asked participants to imagine the existence of a clinical test that could indicate the risk of ASD.

Eight questions were adapted from a survey of attitudes to genetic research and testing in families with a risk of malignant melanoma [9] and adjusted to the context of ASD. The remaining 15 questions were developed by our team and subsequently tested by representatives of the Autism Association Norway, guided by themes elicited in a qualitative study of awareness and attitudes among parents of children with ASD [19]. Overall psychometric properties of the questionnaire have not been formally established, but the development process involved a team of experts from multiple clinical disciplines and non-expert representatives from the advocacy organization. Thus, we consider face and content validity to be high. Responses were designed in a 5-point Likert format with the “alternatives” “strongly disagree”, “partly disagree”, “neither disagree nor agree”, “partly agree” and “strongly agree”, in addition to “Don’t know” and “I don’t have an opinion”. We estimated 10–15 min time to complete the questionnaire.

The results regarding attitudes towards (i) genetic research and (ii) clinical genetic testing have been reported previously [26, 29]. Attitudes towards (iii) information about future health and genetic risk of ASD were addressed in four items and reported here. The collected information was anonymous and the Regional Committee for Medical and Health Research Ethics (REC) concluded that the study did not require REC approval.

### Data handling and analyses

Stata version 16 (StataCorp. 2019. Stata Statistical Software: Release 16. College Station, TX: StataCorp LLC) was used for descriptive statistics and IBM SPSS version 27 was used for *t* tests and regression analyses. The responses from the questionnaire received by post were scanned and read using ABBYY FlexiCapture 10 (ABBYY Europe, Munich, Germany) and imported into an SPSS file. The online responses were imported directly.

First, descriptive statistics was performed. We treated the attitude variables as interval data and used *t* tests to compare the attitudes of (a) mothers versus fathers, (b) parents of children with infantile autism versus parents of children with Asperger syndrome and (c) parents in favour of versus parents against clinical genetic testing [30].

To investigate how parents’ attitudes to benefits and harms of genetic testing influence their attitudes to statements about receiving information relevant for future health and sharing ASD-specific genetic risk information, regression analyses with forward stepwise variable selection were performed. To entertain an empirically guided analysis, each step of the regression analyses was examined in order to identify sets of variables that in combination may represent sets associated with the reported attitude [31]. Due to lack of previous empirical knowledge about attitudes towards genetic testing among parents of children with ASD and a need to reduce model size, a forward stepwise procedure was chosen. Selection criterion for inclusion of variables in the model was *p* < 0.05. The final model selection was evaluated with the Automatic Linear Modelling procedure in SPSS to search the entire space of all possible models for best subset modelling [32]. Akaike’s Information Criterion Corrected (AICC) was used as selection criterion to prevent overfitting [32].

## Results

### Sample description

Demographic and diagnostic characteristics of the responding parents and their children with ASD are shown in Table 1.

**Table 1:** Demographics of parents and their children with ASD.

Responding parents *n* = 1455	
Age of parents: mean (SD) {range}	46.7 (8.6) {22–87}
Gender of parents, females	81%
Age of children with ASD: mean (SD) {range}	16.5 (7.6) {3–58}
Gender of the children with ASD, males	80.5%
Specific ASD diagnoses of the children	
Asperger syndrome	49.4%
Infantile autism	37.6%
Other ASD (Atypical autism and PDD-NOS)	13.0%

*ASD* Autism spectrum disorder, *SD* standard deviation, *PDD-NOS* Pervasive Developmental Disorder-Not Otherwise Specified.

### Attitudes toward sharing genetic risk information and towards knowing about future health

As seen in Tables 2, 74.4% of the parents agreed to the statement “parents should decide whom to inform about genetic risk for ASD” and 41.1% agreed to “inform as many as possible”. A total of 58.0% of the parents agreed to “obligation to know” and 51.7% agreed to “*right not to know*”.

**Table 2:** Parents’ agreement to statements regarding sharing information about genetic risk of ASD and knowing about their own and their children’s future health.

Statement	ASD	*n*, Mean (SD)	Disagree (1–2)	Neither (3)	Agree (4–5)	NA	Test result*
“Parents should decide” who to inform about genetic risk for ASD.	A	456, 4.28 (1.16)					*t*(1052) = −1.182, *p* = 0.237
	AS	598, 4.36 (1.12)					
	A+AS	1380, 4.31 (1.13)	8.9%	6.8%	74.4%	10.0%	
It is important to “inform as many as possible” in the family about genetic risk for ASD.	A	467, 3.28 (1.45)					t(1068) = 2.820, *p* < 0.005
	AS	603, 3.02 (1.46)					
	A + AS	1389, 3.15 (1.45)	31.5%	17.9%	41.1%	9.4%	
Everyone has an “obligation to know” information relevant for future health of oneself and one’s children.	A	489, 3.71 (1.41)					t(1128) = 1.473, *p* = 0.141
	AS	641, 3.58 (1.45)					
	A + AS	1419, 3.65 (1.42)	22.3%	13.5%	58.0%	6.4%	
A “right not to know” information relevant for future health of oneself and one’s children is important.	A	483, 3.51 (1.47)					t(1109) = 0.655, *p* = 0.512
	AS	628, 3.45 (1.44)					
	A + AS	1403, 3.45 (1.46)	26.0%	15.0%	51.7%	7.3%	

A = Infantile autism, AS = Asperger syndrome, SD = Standard deviation. All rows add to 100%, NA = Do not know / Have no opinion. *Independent-samples *t* test, two-tailed.

Significantly more parents of children with infantile autism agreed to the statement “inform as many as possible” in comparison to parents of children with Asperger syndrome (*t*(1068) = 2.820, *p* < 0.05). The effect of disease severity is however small. Both means falls between “neither disagree nor agree” and “partly agree”. This disease severity association was still present after controlling for other parent and child characteristics (i.e., parent’s gender, parent’s age, child’s gender and child’s age) (*β* = −0.091, *t* = −2.897, *p* < 0.05) and also explained a significant proportion of variance in the attitude to “inform as many as possible” (*R*^2^ = 0.011, *F*(5,1038) = 2.36, *p* < 0.05). None of the five parent and child characteristics (i.e., parent’s gender, parent’s age, ASD diagnose of child, child’s gender and child’s age) we collected were significantly associated with the other three statements (*p* values range from 0.057 to 0.952).

As reported earlier 8.59% (*n* = 125) of the parents in our sample were explicitly against clinical genetic testing for ASD [26]. Parents of children with Asperger syndrome (M = 0.11, SD = 0.310) were more frequently against clinical genetic testing than parents of children with infantile autism (M = 0.07, SD = 0.254) (*t*(1223.200) = 2.294, *p* < 0.05). Parents in favour of genetic testing were not significantly different from parents against genetic testing in their response to whether “parents should decide whom to inform”, but they (M = 3.28, SD = 1.411) agreed significantly more than parents against genetic testing (M = 1.76, SD = 1.083) to the statement “inform as many as possible” (*t*(154.015) = 13.856, *p* < 0.05). Parents in favour of testing (M = 3.73, SD = 1.381) also agreed significantly more than parents against testing (M = 2.81, SD = 1.568) to “an obligation to know” (*t*(129.986) = 6.096, *p* < 0.05) and they agreed significantly less (M = 3.42, SD = 1.456) to the statement “*right not to know*” than parents against genetic testing (M = 3.79, SD = 1.454) (*t*(1299) = −2.580, *p* < 0.05).

### Associations with perceived benefits and harms of clinical genetic testing

The independent variables are listed in Table 3. Results of statistical analyses for variables both selected and excluded at each step of the analyses are found in Table 4A. Reports of the identification of close alternatives at each step which may form sets together with the selected variable associated with the outcome variable are found in Table 4B together with the best model from an all-possible-subset approach.

**Table 3:** Independent variables for interest in sharing genetic risk information and knowing about future health.

Variable	Disagree		Neither	Agree		Don’t know / no opinion	*n*	Statement
	Entirely (1)	Partly(2)	(3)	Partly(4)	Entirely (5)			
Treatment relevant	17.83%	9.55%	9.84%	20.67%	32.41%	9.69%	1413	“I would take a genetic test only if it had direct treatment consequences for me or my children.”
Cause explained	7.59%	4.15%	5.56%	20.04%	56.33%	6.33%	1422	“I would take a genetic test if it could help explain the cause of my own or my children’s condition.”
Future treatment	12.89%	7.18%	8.17%	26.90%	36.83%	8.03%	1420	“I would take a genetic test only if it could contribute to better treatment in the future; not necessarily for my own children.”
Understand ASD	4.91%	2.39%	4.56%	20.63%	62.95%	4.57%	1425	“I would take a genetic test if this would contribute to a better understanding of what ASD really is.”
Prevent recurrence	30.66%	7.82%	10.08%	11.42%	28.54%	11.49%	1419	“I would take a genetic test if it provided an opportunity to prevent me from having more children with ASD.”
Family planning	37.90%	15.46%	15.38%	11.93%	12.14%	7.20%	1417	“Genetic testing is important for family planning.”
Improve planning	5.79%	3.91%	7.82%	26.00%	48.81%	7.68%	1381	“An early diagnosis based on genetic testing will improve the opportunities for planning good interventions and facilitations.”
Social construction	30.34%	16.87%	11.44%	16.36%	9.27%	15.72%	1381	“Difficulties due to ASD are mainly socially constructed. Genetic testing will therefore in any case have minimal consequences for interventions and facilitations.”
Insurance discrimination	5.48%	2.74%	7.35%	20.84%	45.93%	17.66%	1387	“A genetic test that shows the risk of developing ASD will lead to discrimination from insurance companies.”
Increased concern	14.16%	10.40%	11.42%	31.79%	24.35%	7.88%	1384	“Early diagnosis based on genetic testing will increase parents’ concern for the child’s future health and development.”
Family conflict	10.37%	7.25%	18.93%	29.30%	19.65%	14.51%	1379	“Genetic testing can lead to family conflicts.”

*5-point agreement scale. Scenario: “Currently no test can predict ASD. Imagine there was a genetic test that could determine risk of ASD. To what extent do you agree or disagree with the following statements?”. All rows add to 100 %.

**Table 4:** **A** Stepwise analyses: “decide who to inform”, “inform as many as possible”, “obligation to know” and “*right not to know*”. **B** Reports of stepwise analyses and best subset models: “decide who to inform”, “inform as many as possible”, “obligation to know” and “*right not to know*”.

A															
		Parents should decide who to inform about genetic ASD risk									Parents should inform as many as possible in the family about genetic ASD risk				
		*p* values									*p* values				
Variable	*r*	0	1	2	3	4	5	6	7	*r*	0	1	2	3	4
Treatment relevant	0.136	<0.001	<0.001	0.003	0.016	0.017	0.032	0.043	»[0.043]	0.075	0.011	0.005	~0.169	0.230	0.422
Explain cause	0.141	<0.001	<0.001	~0.261	0.108	0.053	?0.275	0.123	0.197	0.329	<0.001	0.000	0.001	0.010	»[0.010]
Future treatment	0.111	<0.001	<0.001	~0.264	0.712	0.796	0.855	0.834	0.520	0.135	<0.001	0.001	~0.095	0.279	0.566
Understand ASD	0.208	<0.001	<0.001	»[<0.001]	[<0.001]	[<0.001]	[<0.001]	[<0.001]	[<0.001]	0.271	<0.001	0.000	0.014	~0.114	0.704
Recurrence prevention	0.009	0.772	0.234	0.486	0.930	0.913	0.478	0.303	0.331	0.415	<0.001	0.000	<0.001	»[<0.001]	[<0.001]
Family planning	−0.071	0.015	~0.157	0.004	0.013	0.022	0.003	»[0.003]	[0.004]	0.441	<0.001	»[<0.001]	[<0.001]	[<0.001]	[<0.001]
Improve planning	0.173	<0.001	<0.001	0.008	0.002	<0.001	»[<0.001]	[<0.001]	[<0.001]	0.419	<0.001	0.000	[<0.001]	[<0.001]	[<0.001]
Social construction	0.162	<0.001	<0.001	<0.001	»[<0.001]	[<0.001]	[<0.001]	[<0.001]	[<0.001]	−0.133	<0.001	0.007	0.039	~0.130	0.179
Insurance discrimination	0.270	<0.001	»[<0.001]	[<0.001]	[<0.001]	[<0.001]	[<0.001]	[<0.001]	[<0.001]	−0.054	0.077	0.781	0.100	0.198	0.197
Increased concern	0.144	<0.001	0.001	<0.001	0.002	~0.117	0.086	0.079	0.106	−0.068	0.018	0.121	0.472	0.325	0.354
Family conflict	0.177	<0.001	0.001	<0.001	<0.001	»[<0.001]	[<0.001]	[<0.001]	[<0.001]	−0.118	0.365	0.010	~0.101	0.076	0.104
Adjusted *R*^2^			0.072	0.106	0.125	0.136	0.145	0.152	0.155			0.194	0.294	0.315	0.319

		Everyone has an obligation to know as much as possible relevant for future health of oneself and one’s children								A ‘*right not to know’* information relevant for future health of oneself and one’s children is important				
		*p* values								*p* values				
Variable	*r*	0	1	2	3	4	5	6	*r*	0	1	2	3	4
Treatment relevant	0.120	<0.001	0.010	0.003	0.016	0.010	~0.143	0.138	0.078	0.007	0.019	0.040	~0.070	0.306
Explain cause	0.296	<0.001	<0.001	<0.001	0.042	~0.076	0.097	0.148	−0.035	0.222	0.794	0.664	0.665	0.287
Future treatment	0.200	<0.001	<0.001	<0.001	0.004	0.002	»[0.002]	[0.003]	0.075	0.009	0.018	0.019	0.038	»[0.038]
Understand ASD	0.299	<0.001	<0.001	<0.001	»[<0.001]	[<0.001]	[<0.001]	[0.001]	−0.004	0.882	0.798	0.843	0.824	0.228
Recurrence prevention	0.268	<0.001	<0.001	0.004	0.034	0.027	0.039	»[0.039]	−0.031	0.290	0.561	0.855	0.783	0.541
Family planning	0.286	<0.001	<0.001	»[<0.001]	[<0.001]	[<0.001]	[<0.001]	[<0.001]	−0.080	0.004	~0.065	?0.133	0.132	0.089
Improve planning	0.340	<0.001	»[<0.001]	[<0.001]	[<0.001]	[<0.001]	[<0.001]	[<0.001]	−0.051	0.077	0.426	0.175	0.214	0.093
Social construction	−0.035	0.250	0.967	0.451	0.335	0.143	0.310	0.209	0.085	0.005	~0.085	0.248	0.373	0.485
Insurance discrimination	−0.037	0.223	0.029	~0.190	0.095	0.588	0.672	0.806	0.200	<0.001	<0.001	»[<0.001]	[<0.001]	[<0.001]
Increased concern	−0.110	<0.001	0.007	0.012	0.007	~0.458	0.297	0.266	0.209	<0.001	0.001	0.001	»[0.001]	[0.002]
Family conflict	−0.167	<0.001	<0.001	<0.001	<0.001	»[<0.001]	[<0.001]	[<0.001]	0.235	<0.001	»[<0.001]	[<0.001]	[<0.001]	[<0.001]
Adjusted *R*^2^			0.115	0.157	0.177	0.189	0.196	0.198			0.054	0.073	0.082	0.085

B													
Report of “decide who to inform” stepwise analysis		Best subset* model of “decide who to inform”					Report of “inform as many as possible” stepwise analysis		Best subset* model of “inform as many as possible”				
Step	Variable added	Variable	Coef.	*p*	95 % CI	RVI	Step	Variable added	Variable	Coef.	*p*	95 % CI	RVI
1	Insurance discrimination	Insurance discrimination	0.172	<0.001	0.115–0.229	0.274	1	Family planning	Improve planning	0.336	<0.001	0.269–0.403	0.451
2	Understand ASD	Understand ASD	0.176	<0.001	0.110–0.243	0.213	2	Improve planning	Family planning	0.255	<0.001	0.197–0.313	0.349
3	Social construction	Improve planning	0.131	<0.001	0.072–019.0	0.150	3	Recurrence prevention	Recurrence prevention	0.143	<0.001	0.092–0.194	0.140
4	Family conflict	Social construction	0.100	<0.001	0.055–0.146	0.145	4	Explain cause	Explain cause	0.102	0.002	0.038–0.165	0.045
5	Improve planning	Family planning	−0.071	0.001	−0.114 to −0.027	0.080							
6	Family planning	Family conflict	0.085	0.004	0.028–0.143	0.066							
7	Treatment relevant	Treatment relevant	0.045	0.030	0.004–0.086	0.037							
Others considered: Recurrence prevention.		* Auto data preparation (trim outliers, replace missing values), selection criterion: AICC; Adjusted *R*^2^ = 0.143; *RVI* relative variable importance					Others considered: Insurance discrimination.		* Auto data preparation (trim outliers, replace missing values), selection criterion: AICC; Adjusted *R*^2^ = 0.305; *RVI* relative variable importance				

Report of “obligation to know” stepwise analysis		Best subset* model of “obligation to know”					Report of “*right not to know*” stepwise analysis		Best subset* model of “*right not to know*”				
Step	Variable added	Variable	Coef.	*p*	95 % CI	RVI	Step	Variable added	Variable	Coef.	*p*	95 % CI	RVI
1	Improve planning	Improve planning	0.224	<0.001	0.152–0.296	0.379	1	Family conflict	Insurance discrimination	0.178	<0.001	0.104–0.253	0.350
2	Family planning	Family planning	0.151	<0.001	0.092–0.210	0.255	2	Insurance discrimination	Family conflict	0.159	<0.001	0.082–0.236	0.263
3	Understand ASD	Understand ASD	0.139	0.003	0.047–0.232	0.088	3	Increased concern	Increased concern	0.127	<0.001	0.061–0.193	0.229
4	Family conflict	Family conflict	−0.102	0.003	−0.170 to −0.034	0.087	4	Future treatment	Future treatment	0.067	0.020	0.010–0.123	0.086
5	Future treatment	Future treatment	0.077	0.011	0.017–0.137	0.065			Family planning	−0.058	0.035	−0.113 to −0.004	0.071
6	Recurrence prevention	Recurrence prevention	0.058	0.031	0.005–0.110	0.047							
Others considered: Social construction.		* Auto data preparation (trim outliers, replace missing values), selection criterion: AICC; Adjusted *R*^2^ = 0.190; *RVI* relative variable importance					Others considered: Explain cause, understand ASD, recurrence prevention, Improve planning.		* Auto data preparation (trim outliers, replace missing values), selection criterion: AICC; Adjusted *R*^2^ = 0.078; *RVI* relative variable importance				

*r* Pearson correlation coefficients; » variable selected for entry; ~ identification of close alternative;? weak close alternative (A close alternative is a variable originally significant, later non-significant after selection of some other variable); [] Variable in model; Selection criteria: *p* < 0.05 to enter, *p* > 0.10 to remove. *P* values of all models: <.001.

### Parents should decide whom to share genetic risk information with

In an all-possible-subsets approach of the attitude to the normativity of parental autonomy (“parents should decide”), the model with the smallest information loss was comprised of eight of the 11 predictors with varying relative variable importance (RVI) in the model: ‘insurance discrimination’ (RVI = 0.274), “understand ASD” (RVI = 0.213), “improve planning” (RVI = 0.150), “socially constructed” (RVI = 0.145), “family planning” (RVI = 0.080) “family conflict” (RVI = 0.066), and “treatment relevant” (RVI = 0.037) and “increased concern” (RVI = 0.035); *p* range from <0.001 to 0.036; adj *R*^2^ = 0.143, *p* < 0.001. A forward stepwise regression analysis of “parents should decide” produced a final model with the same variables except from “increased concern” (*p* range from <0.001 to 0.043; adj *R*^2^ = 0.155, *p* < 0.001).

### Share genetic risk information with as many as possible in the family

A best subset regression analysis of the attitude to the importance of full intra-familial disclosure (“inform as many as possible”) produced a model composed of “improve planning” (RVI = 0.451), “family planning” (RVI = 0.349), “recurrence prevention” (RVI = 0.140), “explain cause” (RVI = 0.045) and “family conflict” (RVI = 0.014); *p* range from <0.001 to 0.077; adj *R*^2^ = 0.305, *p* < 0.001. A forward stepwise analysis of “important to inform as many as possible” included the same variables in the final model except from “family conflict” (*p* range from <0.001 to 0.010; adj *R*^2^ = 0.319, *p* < 0.001).

### Obligation to know

In a best subset regression analysis of the attitude to the normativity of being informed (“obligation to know”) nine of the eleven predictor variables was included, the most important being “improve planning” (RVI = 0.379), ‘family planning’ (RVI = 0.255), “understand ASD” (RVI = 0.088), “family conflict” (RVI = 0.087) and “future treatment” (RVI = 0.065) and “recurrence prevention” (RVI = 0.047); *p* range from <0.001 to 0.142; adj *R*^2^ = 0.190, *p* < 0.001. For all predictors, see Table 4B. In a forward stepwise analysis of “obligation to know” the same variables presented from the best subset model were included in the final stepwise model (*p* range from <0.001 to 0.045; *R*^2^ = 0.206, *p* < 0.001).

### A “right not to know”

In a best subset regression analysis of the attitude to the importance of a right to ignorance (“*right not to know*”) “insurance discrimination” (RVI = 0.350), “family conflict” (RVI = 0.263), “increased concern” (RVI = 0.229), “future treatment” (RVI = 0.086) and “family planning” (RVI = 0.071) were included (*p* range from <0.001 to 0.035; adj *R*^2^ = 0.078, *p* < 0.001). In a forward stepwise analysis of “*right not to know*”, the same variables except ‘family planning’ were included in the final model (*p* range from <0.001 to 0.038; *R*^2^ = 0.085, *p* < 0.001).

## Discussion

The main findings of the present study were that a majority of the parents perceive it an obligation to obtain as much information as possible about their own and their children’s future health, and that they want autonomy and intra-familial confidentiality about genetic risk information. Interestingly, only a small majority agreed that a ‘*right not to know*’ is important. These findings could be used to inform future guidelines about procedures for information about genetic testing in ASD.

In support of our hypothesis #1, a main finding was that 74.4% of the respondents agreed that parents themselves should decide whom to inform about a genetic risk of ASD. This attitude was independent of their child’s type of ASD diagnosis. The all-possible-subsets showed that the genetic testing harm ‘insurance discrimination’ contributed significantly to parents’ attitude. This implies that confidentiality in genetic testing for ASD is important. The concern about insurance is unexpected in Norway where the health care system is public and any kind of discrimination on the basis of genetic constitution is prohibited by law [26]. The genetic testing variable ‘understand ASD’ also contributed significantly to this attitude, and relatively more so than the practical benefits and harms, which may indicate that knowledge of the cause of the condition is of primary importance for the parents, perhaps as a sense of relief from uncertainty.

Another main finding was that only 41.1% of the respondents agreed that it is important to inform as many as possible in the family about genetic risk of ASD. This supports hypothesis #2. Parents in favour of clinical genetic testing were indifferent, not positive, to extensive intra-familial disclosure, which may underscore the need for confidentiality even within the family. This seems in accordance with the duty of confidentiality of professionals towards their patients [22]. In the familial sphere this attitude seems to differ from a previously documented altruism towards contributing to genetic research in the current sample [29] and may also be seen to conflict with the focus on solidarity in communal models of ethics [33]. Agreement to extensive intra-familial disclosure was significantly stronger for those with children with increased disease severity. This may indicate inadequate knowledge of inheritance patterns and underscore the importance of genetic counseling as higher heritability is associated with milder ASD subtypes [3, 34]. It may also be explained by a perception of burden of which symptom severity is a predictor [35].

In support of the first part of our hypothesis #3 we found that the majority of parents (58.0 %) agree that everyone has an obligation to know as much as possible relevant for future health of oneself and one’s children. This was positively associated with the testing variables (“understand ASD”, “family planning” and “improved planning of interventions and facilitations” etc.) and negatively associated with the variable “family conflict”. This may indicate a focus on their child’s best interests rather than on their own, in line with a recent finding among parents of paediatric neurology patients [36]. Parents’ attitudes may in some cases come in conflict with the child’s future autonomy and “right not to know” although concern for their children’s best interests may indeed facilitate development of autonomy in other cases [37, 38].

We found that half of the sample (51.7%) agreed that it is important to have a “*right not to know*”, which did not support our hypothesis #4. This result is not in accordance with previous results [25]. A “*right not to know*” seems explicitly to be connected to informational privacy [39]. In the perspective of practical everyday coping, respect for individual privacy may relate to spatial privacy directed to the individual’s sense of self [40] and individual preferences may be a topic for genetic counseling rather than for legislation. A practical perspective of genetic testing with counseling and support may explain the relative infrequent interest in a “right” not to know. A “*right not to know*” was associated with “insurance discrimination”, which may be less relevant in countries with public health insurance. It was also related to instigating “family conflict” and “increased concern” for future health and development, both important aspects of genetic counseling.

The attitudes documented here are from parent members of an advocacy organization. Advocacy communities tend to focus on talents and advantageous traits and that the cognitive styles characteristic of ASD may contribute productively to science and society [41, 42]. If ASD is not considered a disease, but rather a valuable trait, attitudes towards genetic testing and genetic information may be affected. The terminology of risk used in medical genetics clinic and genetics research may be unfortunate since it may downplay positive traits. Furthermore, our use of the “disease severity” diagnostic/symptom criteria from international research terminology [43] may not necessary reflect challenges experienced by individuals on the autism spectrum and their families [44]. In the current paper and in the questionnaire, we have used standard international terminology, while we recommend more neutral terms in line with preferences of the communities, such as condition rather than disorder.

The current study provides new empirical knowledge about attitudes in ASD families toward principles regulating generation and dissemination of genetic risk information. Despite its large size, our sample may not be fully representative of attitudes of ASD parents as it was recruited from an advocacy group. Other limitations are lack of comprehensive demographic information, little information about disease severity and lack of information of other children in the family with an ASD diagnosis or psychiatric conditions, which may have impacted the parents’ attitudes. It is also a limitation that the questions were adapted from a questionnaire about malignant melanoma which, although they were general, may be less suitable for ASD. The data was collected in 2014, but the changes in clinical genetics mainly involve new technical equipment, while clinical genetics services are still underutilized in ASD [45], making our findings relevant to the current state of ASD clinical genetics.

In conclusion, these findings illustrate the complexity of the attitudes towards genetic testing in ASD and underscore the need for comprehensive genetic counseling. The results support current trends advocating to move away from non-directiveness and promote a proactive approach that involve recommendations to serve the patient’s or family’s best interests [46]. Surprisingly, we only found support for the importance of a “*right not to know*” by a small majority (51.7%), which seems at odds with the explicit support in various ethical and legal instruments [22]. It could be discussed if this interest in ignorance should be relaxed in the legislation, as it seems to depend on individual circumstances, which are better dealt with in a genetic counseling setting. Furthermore, the findings support current counseling practices which addresses physical, societal, material and emotional aspects of individual families [47]. Our findings also underscore the need to provide contextual information upon delivery of ASD genetic risk information, in line with findings in general psychiatry [48]. Replication in independent samples is warranted before ethical guidelines relevant to modern genetic testing and counseling in ASD are revised.

## Data Availability

The datasets generated during and/or analyzed during the current study are available in the Open Science Framework repository, https://osf.io/zs3mr/
